# A combination of levobupivacaine and lidocaine for paravertebral block in breast cancer patients undergoing quadrantectomy causes greater hemodynamic oscillations than levobupivacaine alone

**DOI:** 10.3325/cmj.2017.58.270

**Published:** 2017-08

**Authors:** Miroslav Župčić, Sandra Graf, Župčić, Viktor Duzel, Tatjana Šimurina, Livija Šakić, Jurica Fudurić, Jasminka Peršec, Milan Milošević, Zdenko Stanec, Anđelko Korušić, Stjepan Barišin

**Affiliations:** 1 Clinic of Anesthesiology, Reanimatology and Intensive Care Medicine, Clinical Hospital Dubrava, Zagreb, Croatia; 2 “J. J. Strossmayer” Faculty of Medicine, Osijek, Croatia; 3 Clinic of Neurology, Clinical Hospital “Sveti Duh“, Zagreb, and “J. J. Strossmayer” Faculty of Medicine, Osijek, Croatia; 4 Department of Anaesthesia, Barking, Havering and Redbridge University Hospitals NHS Trust, London, United Kingdom; 5 Depatment of Health Studies, University of Zadar; Department of Anesthesiology and Intensive Care Medicine, General Hospital Zadar, Zadar; and “J. J. Strossmayer” Faculty of Medicine, Osijek, Croatia; 6 Clinic of Anesthesiology, Reanimatology and Intensive Medicine, University Hospital “Sveti Duh”, Zagreb, and “J. J. Strossmayer” Faculty of Medicine, Osijek, Croatia; 7 Department of Surgery, General Hospital Karlovac, Karlovac, Croatia; 8 Andrija Štampar School of Public Health WHO Collaborative Centre for Occupational Health, University of Zagreb School of Medicine, Zagreb, Croatia; 9 Clinic for Plastic, Reconstructive and Aesthetic Surgery, Clinical Hospital Dubrava, Zagreb, Croatia

## Abstract

**Aim:**

To test for differences in hemodynamic and analgesic properties in patients with breast cancer undergoing quadrantectomy with paravertebral block (PVB) induced with a solution of either one or two local anesthetics.

**Method:**

A prospective, single-center, randomized, double-blinded, controlled trial was conducted from June 2014 until September 2015. A total of 85 women with breast cancer were assigned to receive PVB with either 0.5% levobupivacaine (n = 42) or 0.5% levobupivacaine with 2% lidocaine (n = 43). Hemodynamic variables of interest included intraoperative stroke volume variation (SVV), mean arterial pressure, heart rate, cardiac output, episodes of hypotension, use of crystalloids, and use of inotropes. Analgesic variables of interest were time to block onset, duration of analgesia, and postoperative serial pain assessment using a visual analogue scale.

**Results:**

Although the use of 0.5% levobupivacaine with 2% lidocaine solution for PVB decreased the mean time-to-block onset (14 minutes; *P* < 0.001), it also caused significantly higher SVV values over the 60 minutes of monitoring (mean difference: 4.33; *P* < 0.001). Furthermore, the patients who received 0.5% levobupivacaine with 2% lidocaine experienced shorter mean duration of analgesia (105 minutes; *P* = 0.006) and more episodes of hypotension (17.5%; *P* = 0.048) and received more intraoperative crystalloids (mean volume: 550 mL; *P* < 0.001).

**Conclusion:**

The use of 0.5% levobupivacaine in comparison with 0.5% levobupivacaine with 2% lidocaine solution for PVB had a longer time-to-block onset, but it also reduced hemodynamic disturbances and prolonged the analgesic effect.

Registration No.: NTC02004834

Regional anesthesia is a technique whereby a local anesthetic is injected near a nerve or the spinal cord in order to block or inhibit the reception of and response to pain and motor stimuli from a certain innervation area (1). Previous studies showed a high success of paravertebral block (PVB) for both anesthesia and postoperative analgesia in breast surgery (2-10). The technique of giving a local anesthetic for PVB distributed on several levels is better than a technique of giving it only at one level (11-13). Although significant hemodynamic changes occur during the application of spinal and epidural anesthesia compared with PVB, there is a paucity of data about hemodynamic effects of different local anesthetics used for PVB (14,15). It has not yet been determined which type of local anesthetic solution has more favorable hemodynamic characteristics. Since the effects of local anesthetics on vasomotor tone are well known, we aimed to evaluate whether there were any relevant differences in hemodynamic stability as the primary outcome and the duration of analgesia as the secondary outcome between two local anesthetic solutions, namely, 0.5% levobupivacaine and a combination of 0.5% levobupivacaine and 2% lidocaine, in PVB for breast cancer surgery (14).

## PATIENTS AND METHODS

This study was a single-center, randomized, controlled, double-blind trial conducted at Clinical Hospital Dubrava, Zagreb, Croatia, between June 2014 and September 2015.

### Patients

The candidates for inclusion were consecutive women with breast cancer scheduled for surgical quadrantectomy with ipsilateral axillary lymph node dissection. The inclusion criteria were age 18-80 years, body weight 50-95 kg, American Society of Anesthesiologists (ASA) physical status I or II, and signed informed consent. The exclusion criteria were coagulation disorders, infection in the areas of intended block application, neuropathy, failed PVB, uncontrolled psychiatric disorders, allergy to medications used in the trial, contraindications for the use of Vigileo / FloTrac system, and valvular diseases or abnormal heart rhythm.

### Method

#### Study flow and double-blinding

The randomization schedule was computer-generated by a free online randomization service (16). Eligible patients were randomly allocated to receive either a thoracic PVB with levobupivacaine and lidocaine (LLG group, n = 43) or levobupivacaine alone (LG group, n = 42). The recruiting anesthesiologists and the anesthesia technicians who prepared the local anesthetic solutions were blinded. The two solutions of local anesthetics were indistinguishable by appearance. The PVB was performed by a blinded anesthesiologist in the pre-operative holding area, while the patients received an infusion of 5-8 mL/kg of crystalloid 0.9% NaCl solution (Pliva, Zagreb, Croatia). After the confirmation of a successful block according to the predefined criteria, the patient was transferred into the operating room. All medical staff in the operating room, post-anesthesia care unit (PACU), and on the surgical ward were blinded to the allocated treatments. After the onset of PVB and general anesthesia induction, the mean arterial pressure (MAP) and other hemodynamic parameters were measured using minimally invasive hemodynamic monitoring (Vigileo^TM^/FloTrac^TM^ system 4.0, Edwards Lifesciences, Irvine, CA, USA) for arterial pulse wave analysis. Fluid replacement was monitored continuously during the surgery, starting immediately after anesthesia induction. Surgical breast quadrantectomy with an ipsilateral axillary lymph node dissection was performed by the same two surgeons with more than 10 years of experience. The blinding was broken after the completion of data analysis.

#### Anesthesia procedure

All patients were premedicated with midazolam (Dormicum^®^, Roche) 0.06 mg/kg intramuscularly, 30 minutes before the operation. Electrocardiographic (ECG), non-invasive blood pressure (NIBP), and peripheral oxygen saturation (SpO_2_) monitoring was started in all patients immediately upon their arrival to the preoperative holding unit. After the skin infiltration with 0.5 mL of lidocaine 2% (Lidocaine®, Belupo, Koprivnica, Croatia), an arterial cannula was placed in the radial artery. With the patient in the sitting position, the thoracic paravertebral spaces Th2, Th3, and Th4 were identified using an ultrasound machine (GE Healthcare Logiq Medical Ultrasound System, Wauwatosa, USA) with an ultrasonic linear probe of 8 Hz. We used a neurostimulator needle (Stimuplex HNS 12, B. Braun Melsungen AG, Germany) with a furnished intensity of 2.0 mA, duration of stimulation of 0.1 ms, and a frequency of 1 Hz, together with the ultrasound, to identify the spinal nerves in the paravertebral space. With an in-plane technique, alongside the stimulated intercostal muscle contractions, we confirmed the position of the neurostimulator needle. At the onset of muscle contractions with current strengths ranging from 0.4 to 0.6 mA, injections with the local anesthetic solution were applied on the ipsilateral side to levels Th2, Th3, and Th4 (7.0 mL per level), respectively (9,17). The solution of 0.5% levobupivacaine (Chirocaine^®^, Abbott Laboratories, Dublin, Ireland) and 2% lidocaine (mixture of 7 mL of 2% lidocaine +14 mL of 0.5% levobupivacaine) was administered to the women in the LLG group, and 0.5% levobupivacaine alone was administered to the LG group. After that, the patients were positioned in the supine position and an experienced, blinded anesthesiologist tested the efficacy of the block using the pin prick test and warm-cold test every 5 minutes up to 30 minutes and the number of affected dermatomes. The onset time of the PVB was recorded as one of the primary analgesic outcome measures. The block was considered unsuccessful if a patient did not have a positive response to the above tests between 15 and 30 minutes after administration of the two local anesthetic solutions and/or if the block was spread to less than four dermatomes. These patients were excluded from the trial and proceeded to general anesthesia. After the successful performance of PVB, patients were moved to the operating room where their vital parameters (ECG, NIBP, and SpO_2_) were further monitored. General anesthesia induction was carried out with 1% propofol (10 mg/mL, Fresenius) at a dose of 2 to 2.5 mg/kg intravenously and vecuronium bromide [Norcuron^®^, Schering – Plough,N.V. Organon, Netherlands] at a dose of 0.08 mg/kg intravenously, with a subsequent insertion of a laryngeal mask (I-Gel supraglottic airway) to maintain the airway. All patients were then ventilated by controlled mechanical ventilation with a volume of 8 mL/kg, frequency of 12 breaths per minute, with a mixture of oxygen and air at 40:60% ratio The maintenance of anesthesia and sedation was performed with a continuous infusion of 1% propofol to keep target values of a bispectral index (BIS) monitoring device A-2000 BIS monitor (Aspect Medical Systems, Newton, MA, USA) in the 45-55 range. Muscle relaxation was achieved with vecuronium bromide 0.1 mg/kg/h using a perfusion pump (B. Braun's Perfusor^®^, Melsungen AG, Germany).

#### Hemodynamic monitoring

The stroke volume variation (SVV), MAP, heart rate (HR), and cardiac output (CO) were measured every 5 minutes during the first 60 minutes from the onset of PVB, then every 15 minutes during the next 60 minutes. If the operation lasted more than 2 hours, the measurements were carried out every 30 minutes. Fluid replacement with crystalloid solution was used to maintain a stable hemodynamic status during the operation and was monitored in equally in all operated patients. In cases where the systolic blood pressure decreased below 100 mm Hg or by ≥30% from the baseline values, with bradycardia (HR<50 beats/min), we applied intravenously 5-10 mg of ephedrine hydrochloride (Ephedrine, Biotika, Prague, Czech Republic) and 0.5 mg of atropine sulfate (Atropine, Biotika) with a fluid bolus (5mL/kg/15 minutes). In cases where the systolic blood pressure increased by ≥30% from the baseline values due to inadequate analgesia, 1 µg/kg of fentanyl (Fentanyl^®^, Janssen, Titusville, NJ, USA) was administered.

#### Postoperative care, pain assessment, and pain treatment

Postoperatively, the patients were taken to the PACU and their vital parameters (ECG, NIBP and SpO2) were recorded every 5 minutes. The hemodynamic monitoring system and the arterial cannula were removed. In case of any postoperative nausea and vomiting or retching, the patients were given 0.1 mg/kg of thiethylperazine (Torecan^®^, Krka, Novo Mesto, Slovenia) intravenously. Once the patients fulfilled the standard discharge criteria, they were transferred to the ward with the instruction of non-invasive monitoring of vital parameters until complete block regression. Postoperative intensity of pain was evaluated using an 11-point visual analogue scale (VAS; 0 = no pain; 10 = maximal pain). VAS scores were recorded by trained medical staff every 10 minutes while the patients were in the PACU. Once the patients were transferred to the ward, VAS scores were recorded every 3 hours for 24 hours after the operation. For moderate postoperative pain (VAS score from 3 to <6), the patients received 75 mg of diclofenac sodium (Voltaren, Pliva) intravenously in 100 mL of 0.9% NaCl over 15 minutes. For severe postoperative pain (VAS score ≥6), the patients received a combination of 75 mg of diclofenac sodium intravenously and 100 mg of tramadol hydrochloride (Tramal®7, Herds) in 500 mL of 0.9% NaCl over 30 minutes. The total amount of administered postoperative analgesics was recorded at 12 and 24 hours postoperatively.

#### Outcome measures

The primary hemodynamic outcome was the SVV over the first 60 minutes after the induction of PVB, and the primary analgesic outcomes were time-to-block onset and duration of analgesia. Secondary hemodynamic outcomes were HR, CO, MAP, episodes of hypotension, use of crystalloids, and the use of inotropes. The secondary analgesic outcome was a serial VAS pain assessment.

### Statistical analysis

We assumed that a relative SVV difference between time over treatment in SVV of 60% would be practically and clinically relevant and calculated the sample size and power of the study for repeated measures ANOVA under following conditions: power 95%, 13 repeated measurements, 2 treatment groups, alpha 0.05, autocorrelation 0.5, and effect size 0.3 (assuming that the reference mean was 1, tested mean was 1.6, and SD within each group was 1). The estimated sample size was 80 subjects, or 40 subjects per group. GPower for Windows, version 3.1.3, was used for data analysis.

Kolmogorov-Smirnov test was used to analyze the distribution of quantitative data. Socio-demographic indicators and indicators of analgesia were analyzed using appropriate non-parametric tests, while the hemodynamic values were analyzed using parametric tests. The comparisons between the quantitative values were assessed using the Mann-Whitney U test. The differences in categorical values were analyzed with Fisher exact test. A two-way repeated measured analysis of variance (RM-ANOVA) was conducted to test the hypothesis that there were no changes in primary hemodynamic (SVV) and secondary hemodynamic parameters (MAP, HR and CO) over the first 60 minutes regarding time and treatment over time interaction. Maulchy’s test of sphericity was performed to determine the appropriate correction of within-subject effects. Because there were no sphericity assumed in any of RM-ANOVA tests, the Greenhouse-Geisser correction was used to alter the degrees of freedom and produce an F-ratio where the type I error rate is reduced. Serial (repeated-measures) VAS scores were also analyzed by RM-ANOVA with VAS data previously ln (natural logarithm)-transformed. Overall and time-specific treatment differences were generated with adjustment for multiplicity using Bonferroni correction (hemodynamic outcomes significance was adjusted to 13 with the level of statistical significance <0.05/13 = 0.0038, and for VAS score to nine repeated pair-wise comparisons with the level of statistical significance <0.05/9 = 0.0055). In all other cases, *P* < 0.05 was considered significant. Statistical analyses were performed using the IBM SPSS Statistics for Windows, version 21.0 (IBM Corp., Armonk, NY, USA).

## RESULTS

**Figure 1:**
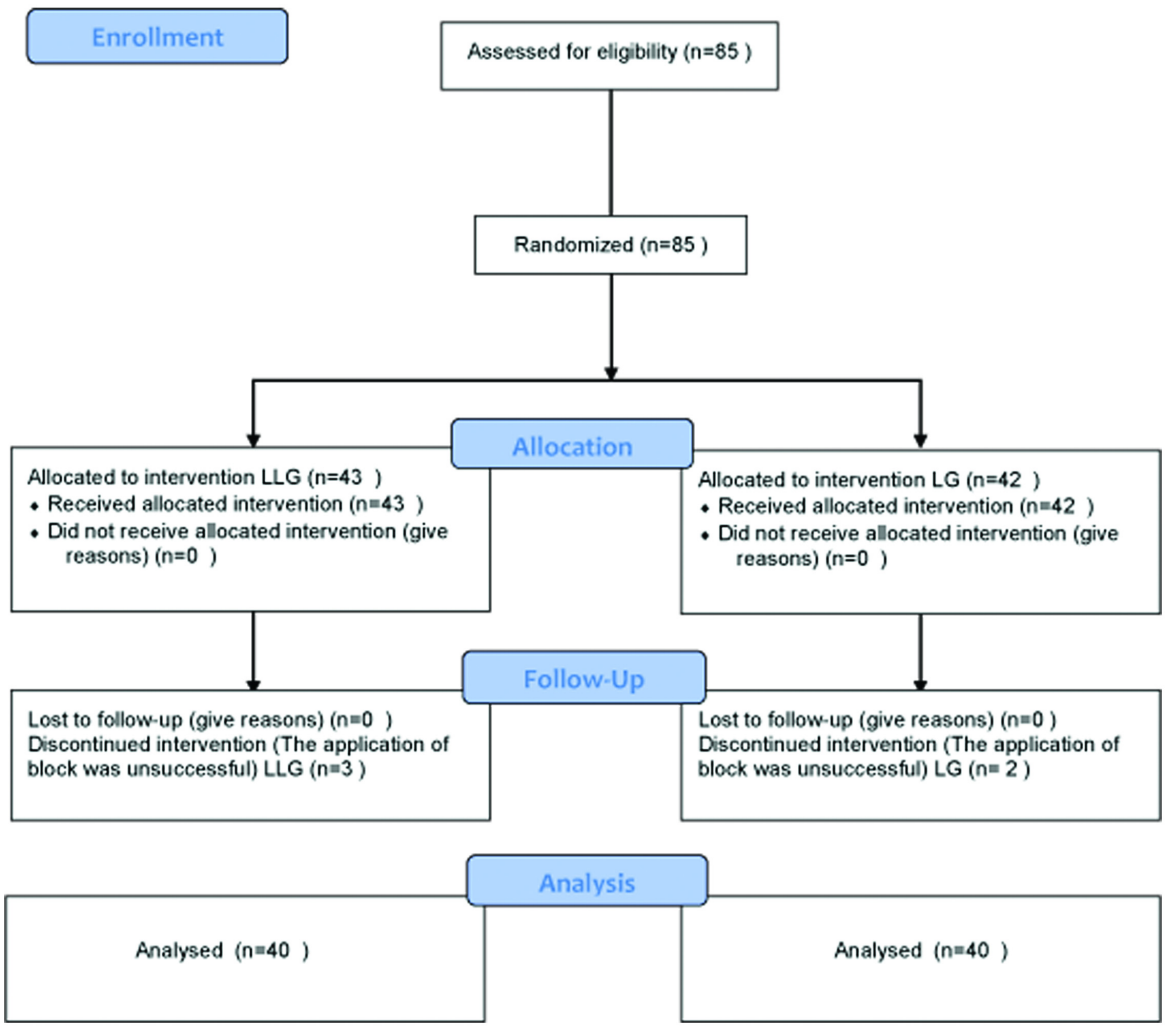
Flow diagram of breast cancer patients included in the trial.

A total of 85 patients were included in the trial. From them, 5 patients (3 from the LLG group and 2 from the LG group) were excluded due to failed PVB (Figure 1). Statistical analysis of patient characteristics, duration of surgery, and mobilization after surgery included Mann Whitney U test and Fisher test and did not show significant differences between groups (Table 1)

**Table 1:** Characteristics of breast cancer patients receiving paravertebral block, duration of surgery, and mobilization after surgery*

Characteristics	No. (%) of patients	
	LG group (n = 40)	LLG group (n = 40)
Age (years; median, 95% CI)	54.5 (50.0-60.0)	60.0 (55.0-67.0)
BMI (kg/m^2^; median, 95% CI)	24.6 (23.1-26.2)	26.2 (24.3-28.0)
Tumor localization: right breast	20 (50.0)	22 (55.0)
ASA PS 2	23 (57.5)	30 (75.0)
HA	13 (32.5)	17 (42.5)
Duration of surgery (minutes; median, 95% CI)	50.0 (50.0-60.0)	50.0 (50.0-60.0)
Mobilization after surgery (minutes; median, 95% CI (min)	240.0 (220.0-270.0)	205.0 (180.0-260.0)

*Abbreviations: LG – 0.5% levobupivacaine; LLG – 0.5% levobupivacaine + 2% lidocaine; CI –confidence interval; BMI – body mass index; ASA – American Society of Anesthesiologists physical status; HA –arterial hypertension.

### Primary and secondary hemodynamic outcomes

**Figure 2:**
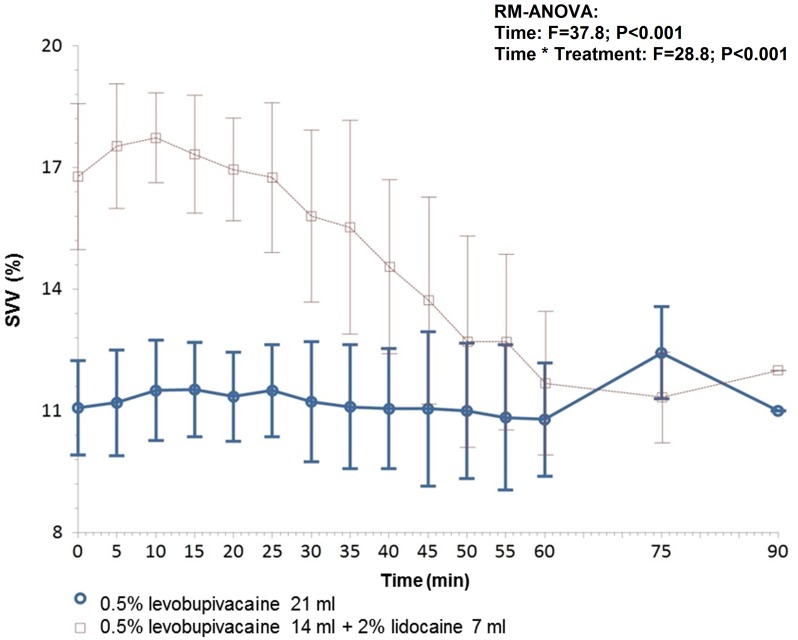
The differences in the dynamics of stroke volume variation (SVV, %) between the test groups and the times of measurement according to the analysis of a variance for repeated measurements (0 denotes the initial hemodynamic values measured by Vigileo/FloTrac system after a successful block performance). Error bars represent mean values (95% confidence interval).

**Figure 3:**
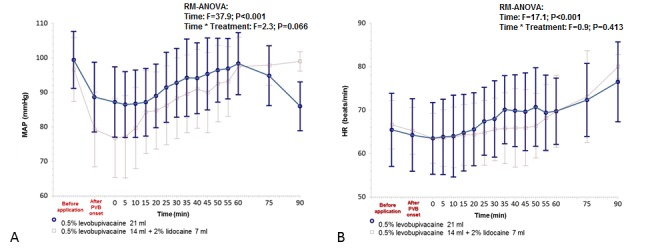
The differences in the dynamics of (**A**) mean arterial pressure (MAP, mmHg) and (**B**) heart rate (HR, bpm) between the test groups and the times (minutes) of measurement according to the analysis of a variance for repeated measures (0 indicates the initial values measured by hemodynamic Vigileo / FloTrac system after performing a successful block). Error bars represent mean (95% confidence interval).

**Figure 4:**
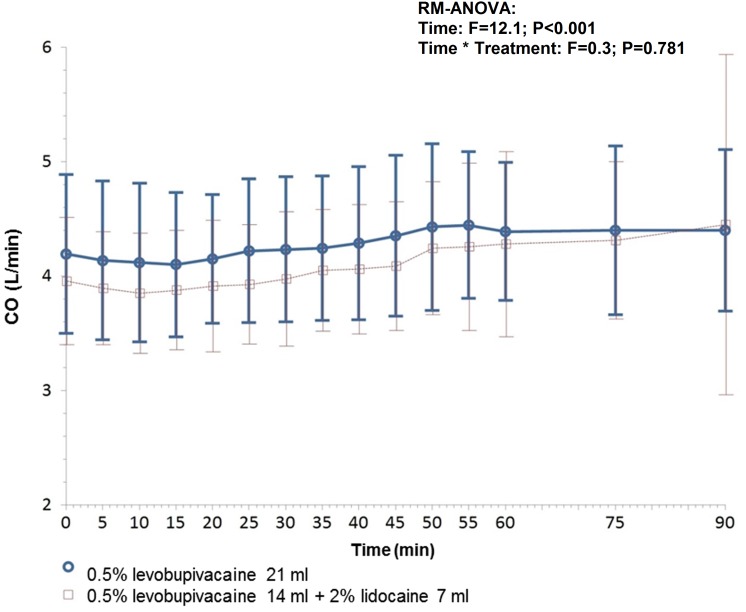
The differences in the dynamics of cardiac output (CO, L/min) between the test groups and the times of measurement according to the analysis of a variance for repeated measurements (0 denotes the initial hemodynamic values measured by Vigileo/FloTrac system after a successful block performance). Error bars represent mean (95% confidence interval).

A two-way between-groups analysis of variance was conducted to explore the impact of treatment over the time (Figures 2, 3, and 4; Table 2). The interaction effect between treatment groups and time was statistically significant only in SVV (F = 28.8; *P* < 0.001, Figure 2) indicating that there were significantly higher SVV values in LLG group during the 60 minutes of monitoring. The main significant difference between the groups occurred in the first 35 minutes (Figure 2). These data were also confirmed with the estimated least-square (LS) means of difference showing a significantly higher value of SVV in the LLG group during the 60 minutes of monitoring after the onset of PVB (*P* < 0.001) regarding time and treatment over time interaction (Table 2). There were no significant differences in MAP, HR, and CO regarding treatment over time interaction. However, hemodynamic changes were significant over the monitored time in all investigated parameters including SVV, MAP, HR, and CO in both treatment groups (Figures 2, 3, and 4.) Between the groups, there was a significant difference in the intraoperative fluid requirement of crystalloid infusion. The LLG group required more intraoperative fluid administration in comparison to the LG group (Table 3). Also, the incidence of hypotension was more common in the LLG group. There were no significant differences between groups in the use of ephedrine hydrochloride and atropine sulfate.

**Table 2:** Primary and secondary hemodynamic outcomes measured by minimally invasive hemodynamic monitoring (Vigileo/FloTrac system) in breast cancer patients receiving paravertebral block: differences between treatment groups in estimated means over the time*

Hemodynamic outcomes	LS means (SE)		LS mean difference (95% CI)	*P^†^*
	LG group (n = 40)	LLG group (n = 40)		
**Primary**				
Overall SVV (%) over 60 minutes	11.13 (0.19)	15.47 (0.20)	4.33 (3.80-4.88)	<0.001
**Secondary**				
Overall MAP (mmHg) over time	90.42 (1.63)	90.01 (1.72)	-0.41 (-5.18-4.37)	0.864
Overall HR (bpm) over time	66.17 (1.24)	66.75 (1.32)	0.58 (-3.05-4.21)	0.750
Overall CO (L/min) over 60 minutes	4.18 (0.10)	4.05 (0.11)	-0.13 (-0.43-0.18)	0.414

*Abbreviations: LS – least square; SE – standard error; LG – 0.5% levobupivacaine; LLG – 0.5% levobupivacaine + 2% lidocaine; CI – confidence interval; SVV – stroke volume variation; MAP – mean arterial pressure; HR – heart rate; CO – cardiac output.

†RM-ANOVA.

**Table 3:** Intraoperative sedation, primary analgesic outcomes, and other secondary hemodynamic outcomes in the operated breast cancer patients receiving paravertebral block*

Outcomes	No. (%) of patients		*P*
	LG group (n = 40)	LLG group (n = 40)	
**Intraoperative sedation**			
Continuous doses of 1% propofol (µg/kg/min; median, 95% CI)	65.0 (60.0-70.0)	70.0 (70.0-80.0)	0.088**^†^**
**Primary analgesic outcomes**			
Time-to-block onset (minutes; median, 95% CI)	37.0 (36.0-40.0)	23.0 (20.0-25.0)	<0.001**^†^**
Duration of analgesia (minutes; median, 95% CI)	490.0 (460.0-520.0)	385.0 (350.0-460.0)	0.006**^†^**
**Other secondary hemodynamic outcomes**			
Intraoperative crystalloids (mL; median, 95% CI)	600.0 (600.0-700.0)	1150.0 (1000.0-1300.0)	<0.001**^†^**
Episodes of hypotension	2 (5.0)	9 (22.5)	0.048^‡^
Ephedrine hydrochloride, 5 mg iv.	2 (5.0)	7 (17.5)	0.154^‡^
Atropine sulfate, 0.5 mg iv.	0 (0)	3 (7.5)	0.241^‡^

*Abbreviations: LG – 0.5% levobupivacaine; LLG – 0.5% levobupivacaine + 2% lidocaine; CI – confidence interval.

†Mann-Whitney U test.

‡Fisher exact test.

### Intraoperative sedation and primary and secondary analgesic outcomes

There were no significant differences in the quantity of continuous infusion of propofol 1% between the groups (Table 3). The values of time from PVB administration to block onset and the duration of postoperative analgesia were both significantly lower in the LLG group than in the LG group. VAS scores at rest showed a significant increase 3 hours after the surgery in the LLG group in comparison with those in the LG group, after which they became the same in both groups (Table 4). The significant changes in VAS score regarding time were confirmed by RM-ANOVA analysis (F = 31.0; *P* < 0.001), but there was no significant difference in time over treatment interaction (F = 2.1; *P* = 0.069). Also, there were no differences in VAS scores between treatment groups in estimated means over the time (Table 4). Between the groups, there was a significant difference only in the amount of administered diclofenac sodium, as it was more frequently administered during the 12 hours postoperatively in the LLG group than in the LG group (11/40 vs 4/40, respectively (*P* = 0.045). There was no need for additional administration of opioids in either group.

**Table 4:** Visual analogue scale scores at rest before the operation, after the operation (at each time point), and throughout the 24-hour measurement period in breast cancer patients receiving paravertebral block*

VAS measurement timing	Median (95% CI) VAS score		*P*
	LG group (n = 40)	LLG group (n = 40)	
Before operation	1 (1.0-2.0)	1 (1.0-1.0)	0.270**^†^**
3 h postoperative	1 (1.0-2.0)	2 (2.0-4.0)	0.039**^†^**
6 h postoperative	2 (2.0-3.0)	2 (2.0-4.0)	0.286**^†^**
9 h postoperative	2 (2.0-4.0)	2 (2.0-4.0)	0.425**^†^**
12h postoperative	2 (2.0-4.0)	2 (2.0-4.0)	0.462**^†^**
15 h postoperative	2 (2.0-4.0)	2 (2.0-4.0)	0.544**^†^**
18 h postoperative.	2 (2.0-4.0)	2 (2.0-4.0)	0.580**^†^**
21 h postoperative	2 (2.0-4.0)	2 (2.0-4.0)	0.560**^†^**
24 h postoperative	2 (2.0-4.0)	2 (2.0-5.0)	0.574**^†^**
over 24 h (ln values; LS means, SE)	0.60 (0.03)	0.63 (0.03)	0.595^‡^

*Abbreviations: VAS – visual analogue scale; CI – confidence interval; LG – 0.5% levobupivacaine; LLG – 0.5% levobupivacaine +2% lidocaine; ln – natural logarithm; LS – least square, SE – Standard error.

**†**Mann-Whitney U test.

‡RM-ANOVA.

### Perioperative complications and characteristics of PVB

There were no significant differences between the groups in the incidence of nausea, dizziness, and bradycardia (Table 5).

**Table 5:** Perioperative complications of paravertebral block in breast cancer patients*

Perioperative complications	No. (%) of patients		*P^†^*
	LG group (n = 40)	LLG group (n = 40)	
Nausea	1 (2.5)	2 (5.0)	0.999
Dizziness	4 (10.0)	4 (10.0)	1
Bradycardia	0 (0.0)	3 (7.5)	0.242

*Abbreviations: LG – 0.5% levobupivacaine; LLG – 0.5% levobupivacaine + 2% lidocaine.

†Fisher exact test.

Significantly lower values were noted in the LLG group regarding the time of onset of sensory block and regression of sensory block (Table 6). Accordingly, the times from PVB initiation to the first hemodynamic measurements were significant lower in the LLG group than in the LG group. The time between PVB application and surgical incision in the LLG group was significantly shorter than in the LG group (Table 6).

**Table 6:** Perioperative characteristics of paravertebral block in breast cancer patients*

Characteristics of PVB	Median (95% CI) time in minutes		*P^†^*
	LG group (n = 40)	LLG group (n = 40)	
TSB onset	8.5 (8.0-10.0)	3.0 (3.0-4.0)	<0.001
TSR end	490.0 (460.0-520.0)	385.0 (350.0-460.0)	0.007
Time from PVB administration to the first hemodynamic measurement	45.0 (45.0-48.0)	30.0 (30.0-35.0)	<0.001
Time from PVB administration to incision	50.0 (50.0-55.0)	35.0 (35.0-40.0)	<0.001

*Abbreviations: CI – confidence interval; LG – 0.5% levobupivacaine; LLG – 0.5% levobupivacaine + 2% lidocaine; TSB – time of sensory blockade; TSR – time of sensory regression; PVB – paravertebral block .

†Mann-Whitney U test.

## DISCUSSION

The results of this trial showed that, for PVB, the administration of a single local anesthetic levobupivacaine in comparison with a solution of two different local anesthetics, levobupivacaine and lidocaine, had a more favorable hemodynamic and analgesic profile. We decided to investigate these two types of local anesthetics because these are the most commonly used local anesthetics in our setting. The administration of one or two local anesthetics is equally acceptable for regional anesthesia, but mixing local anesthetics may offer an advantage of having both fast onset from lidocaine and a long-lasting effect of levobupivacaine (9,13). Many authors advocate that anesthesia for breast surgery can be carried out either under PVB alone or in combination with general anesthesia (2,6-9). Accordingly, a recent meta-analysis by Schnabel et al (9) showed that PVB, alone, or in combination with general anesthesia, results in better postoperative analgesia with fewer adverse effects in comparison with other analgesic methods, such as postoperative rescue opioids or continuous infiltration via wound catheter infusion. Due to a high level of stress in breast cancer patients, we decided to combine PVB with general anesthesia in this trial (2). Although research has shown a high success of PVB in anesthesia and analgesia for breast surgery, it still remains unclear how the PVB affects hemodynamic variables with regard to the type and volume of administered local anesthetic solutions (2,4,5). Even though there are only few studies that mention the complications associated with ultrasound-guided PVB, the value of the ultrasound in the visualization of anatomical structures and in reducing complications is undeniable (18). Using the “in-plane” ultrasound technique (visualization of the entire needle), we increased the degree of precision and managed to use smaller volumes (7 mL per level) of local anesthetic solutions on multiple levels, thereby attempting to avoid communication with the epidural space as much as possible (10-13). A neurostimulator device enabled us to be even more precise, thereby decreasing the risk of possible nerve damage to the spinal nerves (6). Numerous studies showed an increased efficacy of PVB when applied on more levels, with fewer side effects (4-6,9,11-13). Lemay et al (12) reported that the administration of PVB on multiple levels did not lead to increased absorption of local anesthetics. Using the described methods, the failure rate of PVB was 5.9% in this study and between 6.1% and 10.1% in other studies, which may have resulted from differences in technique (19,20). The continuous propofol 1% infusion was adjusted according to BIS values in an attempt to alleviate the incidence of delirium and possible postoperative cognitive decline (21). Due to the variability of volatile anesthetics effect on BIS values, we decided to use intravenous anesthetics for the maintenance of anesthesia, because they have a more uniform effect on BIS values (22,23).We decided to conduct hemodynamic monitoring with the Vigileo/FloTrac system based on the previous studies on surgical patients (24-26). This device system also allowed us to gain reliable data to define volume replacement and response to the same through changes in vessel tone measured by SVV (25,27). SVV represents a dynamic parameter showing the changing effect of intrathoracic pressure on venous return (24). It is also a good indicator and predictor of intravascular volume (24,26,28). Accordingly, Slagt et al (25) have confirmed the pivotal role of SVV as a dynamic parameter in over 85% of studies. Also, SVV is affected by the changes of intrathoracic pressure induced by mechanical ventilation. In order to precisely monitor this parameter, the patient should be mechanically ventilated with a tidal volume of 8 ml/kg along with having a sinus rhythm on the ECG (29). The LLG group in our study had significantly higher SVV values from the beginning of measurement and in all subsequent measurements during the first hour. This may have occurred due to the shorter time required for the complete onset of the block, faster resorption, and stronger vasodilating effects of the solution with two local anesthetics in the LLG group. Thus, in the LLG group, measurements of the hemodynamic parameters (SVV, MAP, HR, CO) with the Vigileo/FloTrac system, commenced at a significantly earlier time compared to the LG group. During the first 60 minutes of SVV measurement, the hemodynamic values of SVV in the LG group were relatively stable and without significant differences within the group, which can be attributed to the longer time to a complete onset of block and slower resorption and vasodilation effect of the local anesthetic. After 60 minutes, there were no statistically significant differences in SVV between the groups due to the adequate volume replacement and correction of hemodynamic derangements. We noted a significantly larger requirement for intraoperative fluid volume replacement in the LLG group in comparison with the LG group. The reasons for this are the previously mentioned characteristics of PVB, which have resulted in higher values of SVV and lower values of MAP in the LLG group. With intraoperative crystalloid infusions for volume replacement, we attained a positive trend in the increase of MAP and a decrease in the variation of SVV between groups. Analogously, there was a decreased requirement for vasoactive agents in the LG group compared with the LLG group. Therefore, we can conclude that SVV, as a dynamic parameter, has a significant role in clinical practice in the regulation and response to the administration of intravenous fluids and vasoactive agents. MAP and HR before and after the PVB onset were measured with non-invasive methods, while in the operating room, they were measured with Vigileo/FloTrac system. The reason was that we wanted to be certain that the block was successful before using minimally invasive hemodynamic monitoring. To increase measurement precision, the measurement intervals were set at 5 minutes during the first hour, while in other studies, measurements were set at 15-minute intervals during the first hour (3,30). Other trials also showed a significant decrease in MAP values between 30 and 45 minutes from block administration (3,30). Garutti et al (30) explained the hypotension as due to the prompt resorption and vasodilating effect of lidocaine and the unrecognized hypovolemic state of the patient. Our trial confirmed the results published by previous studies regarding the hemodynamic changes, namely the increase in SVV, decrease in MAP and CO, and minimal changes in HR (30,31). Casati et al (32) attributed such changes to the effect of PVB on the peripheral and unilateral nerve blockade with resulting local vasodilation and a minor sympathetic block. These effects primarily depend upon the used technique and type of local anesthetic. Previous studies, which investigated the effects of PVB on hemodynamics and used continuous hemodynamic monitoring, were conducted in patients undergoing lung surgery (30,33,34). During these operations, the PVB was not sufficient to provide complete intraoperative analgesia and therefore, the authors needed to supplement analgesia with significant quantities of opioids, thereby directly affecting hemodynamic changes (30,33). Since we did not need to use opioids intraoperatively, the effects that the local anesthetic may have on hemodynamics were more pronounced. The prolonged analgesic effect in the LG group resulted in a significant reduction of postoperative non-steroidal anti-inflammatory drug (NSAID) administration during the first 12 hours postoperatively. Thus we have demonstrated that the administration of a solution with a single local anesthetic had a better analgesic effect. Investigations comparing the combination of PVB and general anesthesia with general anesthesia alone in patients undergoing breast surgery showed a significant reduction in opioid consumption, along with lower VAS pain scores during the first 24 hours postoperatively in the group where PVB was administered (35,36). PVB administration had enabled mobilization in all patients in our study while maintaining the analgesic effects of the block. Considering that both groups reacted well to the postoperative administration of NSAIDs, there was no need for opioid administration. In this manner and according to previously published research, the incidence of postoperative nausea and dizziness associated with opioid administration was reduced (2,7,8).

Our study has several limitations. The technique of choice in this trial requires a profound knowledge of human anatomy and ultrasound techniques. The application of continued postoperative analgesia via a perineural catheter would provide better analgesia and improve patient satisfaction. Measurement of hemodynamic parameters with the FloTrac/Vigileo system was conducted after a confirmation of a successful block and therefore, we were not able to measure SVV and CO before and during the block performance. The usage of the FloTrac/Vigileo system in this setting requires a mechanically ventilated patient under general anesthesia (27). The assessment of SVV with the FloTrac/Vigileo system has not yet been investigated by direct comparison with other techniques. Our patients were hypotensive at baseline because of pre-operative fasting, application of PVB, and anesthesia with propofol. However, it seems likely that the present setting is close to most common clinical situations, with relative hypovolemia as one of the major causes of arterial hypotension.

The administration of a solution with a single local anesthetic, such as 0.5% levobupivacine, for PVB has a longer time-to-block onset in comparison with a solution of two local anesthetics, 0.5% levobupivacaine with 2% lidocaine, along with a reduced impact on hemodynamic changes and a prolonged analgesic effect.
